# Draft Genome Sequence of Thermosipho globiformans Strain MN14

**DOI:** 10.1128/MRA.01728-18

**Published:** 2019-02-28

**Authors:** Kensuke Igarashi

**Affiliations:** a Bioproduction Research Institute, National Institute of Advanced Industrial Science and Technology (AIST), Sapporo, Japan; Broad Institute of MIT and Harvard University

## Abstract

Thermosipho globiformans strain MN14, a Gram-negative, heterotrophic, thermophilic bacterium, was isolated from a hydrothermal vent in the western Pacific Ocean. Here, I present the 1.9-Mb draft genome sequence of the strain, which consists of 75 contigs with 30.8% GC content and 1,900 predicted protein-coding genes.

## ANNOUNCEMENT

Thermosipho globiformans strain MN14 (1), also known as JCM 15059 and DSM 19918, is an anaerobic rod-shaped thermophile belonging to the order *Thermotogales* (2). With the use of a specially developed device, the bacterium was isolated from a hydrothermal vent at the Suiyo Seamount, Izu-Bonin Arc, in the Western Pacific Ocean of Japan, after *in situ* cultivation at interfaces between hot and anaerobic hydrothermal fluid and cool and aerobic seawater above the seabed (1, 3). Although the strain is an anaerobe, it can survive under suboxic conditions and consume oxygen (1, 4), indicating the presence of oxidative stress defense mechanisms, such as those found in other members of *Thermotogales* (5). Additionally, its rapid growth and ability to produce hydrogen from organic materials indicate the potential of this strain for use in industrial applications, such as for biogas production under aerobic gas phase conditions (4). The genome of the strain was sequenced to increase understanding of its phylogeny.

Thermosipho globiformans strain MN14 was provided by the Japan Collection of Microorganisms. The strain was grown on Thermococcus (Tc) medium (6) at 68°C. Genomic DNA was extracted from the stationary-phase culture using a Wizard genomic DNA purification kit (Promega, Madison, WI, USA). The extracted DNA was used with the TruSeq DNA PCR-free library prep kit (Illumina, San Diego, CA, USA), according to the manufacturer’s instructions, to generate Illumina shotgun paired-end (2 × 150 base pair) sequence libraries, which were sequenced on an Illumina HiSeq 2000 platform, yielding 7.32 Gb raw data. The obtained reads were quality trimmed with Trimmomatic v0.33 (7) (SLIDINGWINDOW:6:30, MINLEN:78, and other parameters by default) and then assembled *de novo* using SPAdes v3.12.0 (8) (kmers of 21, 33, 41, 55, and 77 nucleotides [nt] and other parameters by default), resulting in 75 contigs with a total length of 1,934,731 base pairs, a G+C content of 30.8%, and sequencing coverage of 1,579-fold. An *N*_50_ value of 122,000 base pairs was obtained. Automated annotation of the assembled contigs was performed using Prokka v1.13 (9) with default parameters. CRISPRs were detected by MinCED (https://github.com/ctSkennerton/minced). The draft genome contains a total of 1,953 genes (1,900 protein-coding genes), 48 tRNA genes, 4 rRNA genes, 1 transfer-messenger RNA (tmRNA) gene, and 6 CRISPR sequences.

The draft genome sequence of the strain contained genes encoding NADH-rubredoxin oxidoreductase, rubredoxin, rubrerythrin, and peroxiredoxin, while no cytochrome genes were found. These results suggest that the strain is involved in oxidative stress in a similar manner as reported for Thermotoga maritima (10, 11). Further genetic research, including transcriptome analysis, may provide insight into the defense strategy of this strain against oxidative stress. Additionally, the development of a gene modification system based on the draft genome revealed by this study may help expand the industrial utilization of this strain.

### Data availability.

The genome sequence has been deposited in DDBJ/ENA/GenBank under the accession number BHXD00000000. The raw sequencing reads have been deposited in the DDBJ Sequence Read Archive under the accession number DRA007454. The version described in this paper is the first version.
